# Supplementary material for: Intrinsic timing, not temporal prediction, underlies ramping dynamics in visual and parietal cortex during passive behavior
Source: Sci Adv. 2026 Aug 21;12(34):eaed6417. doi: 10.1126/sciadv.aed6417 (PMC13496207; doi:10.1126/sciadv.aed6417)
Supplement: Supplementary file 1 [file sciadv.aed6417_sm.pdf]

Science Advances
AAAS

Supplementary Materials for

Intrinsic timing, not temporal prediction, underlies ramping dynamics in visual and parietal cortex during passive behavior

Yicong Huang *et al.*

Corresponding author: Farzaneh Najafi, fnajafi3@gatech.edu

Sci. Adv. **12**, eaed6417 (2026)
DOI: 10.1126/sciadv.aed6417

This PDF file includes:

Figs. S1 to S10
References

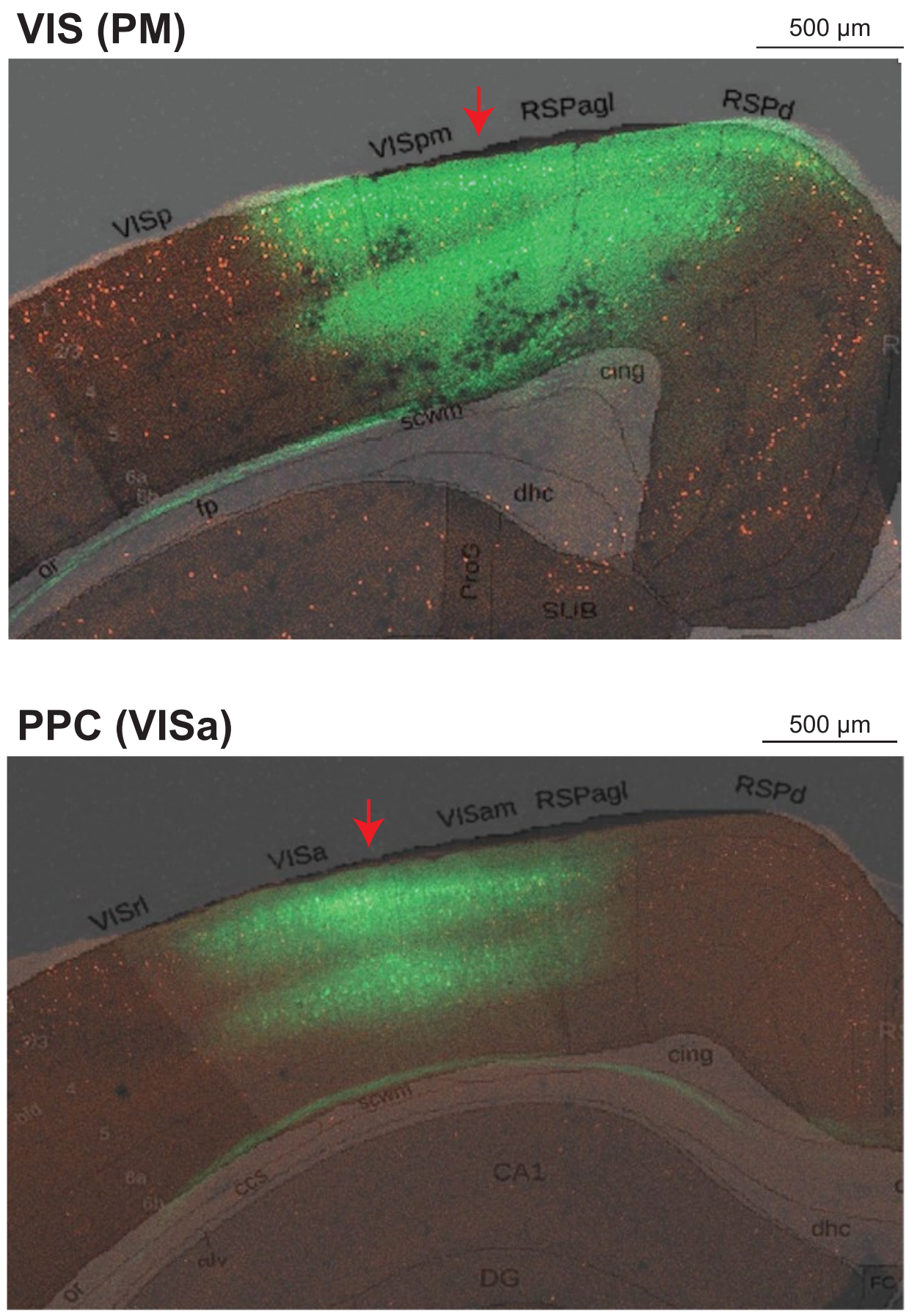

Fig. S1. Histological verification of imaging sites in the medial visual and posterior parietal cortex.

Histology images from two example mice (VIP-tdTomato) imaged in VIS (**top,** area PM) or PPC (**bottom**), with Allen coronal slices superimposed. Red arrow: center of craniotomy window used for imaging, marking VIS (PM), and PPC (VISa). Green: cells labeled by GCaMP8f injection. Red: VIP neurons. Multiple injections were performed within the 3-mm cranial window. Two-photon imaging was primarily conducted from the center of the imaging window.

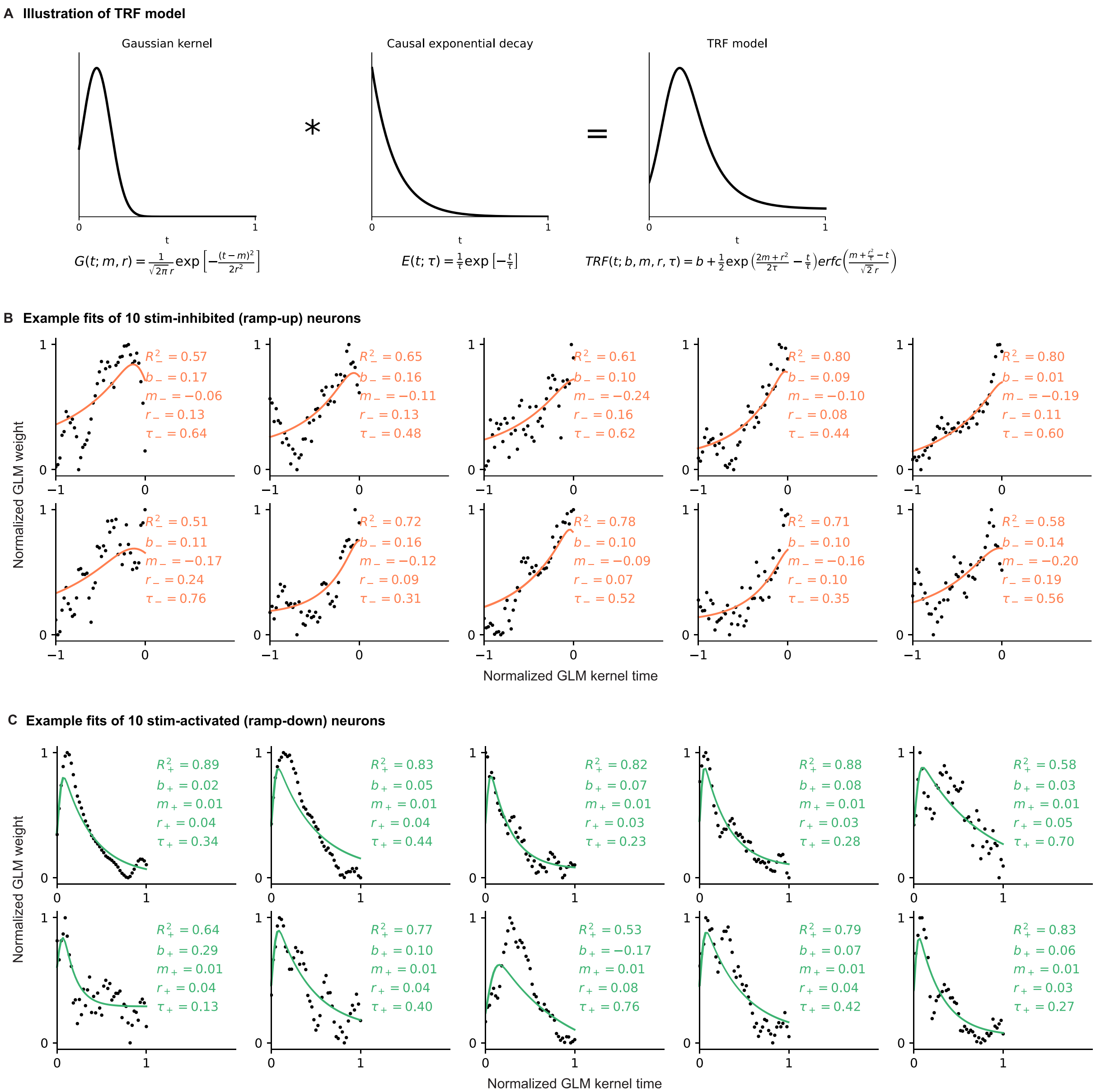

Fig. S2. Example fits of GLM kernels using the TRF model. In all panels, legends report goodness-of-fit (R^2), and model parameters: baseline (b), latency (m), temporal width (r), and time constant (τ), for each ramp-up (-) and ramp-down (+) component.

(A) TRF model function is the convolution results between a Gaussian kernel and a causal exponential function.

(B) Ten examples of GLM kernel components fit by the ramp-up model. Goodness-of-fit values and model parameters are indicated. Dots: normalized GLM kernel weights before time 0. Green: ramp-up TRF model fit.

(C) Same as **(A)**, but for components fit by the ramp-down model. Orange: ramp-down TRF model fit.

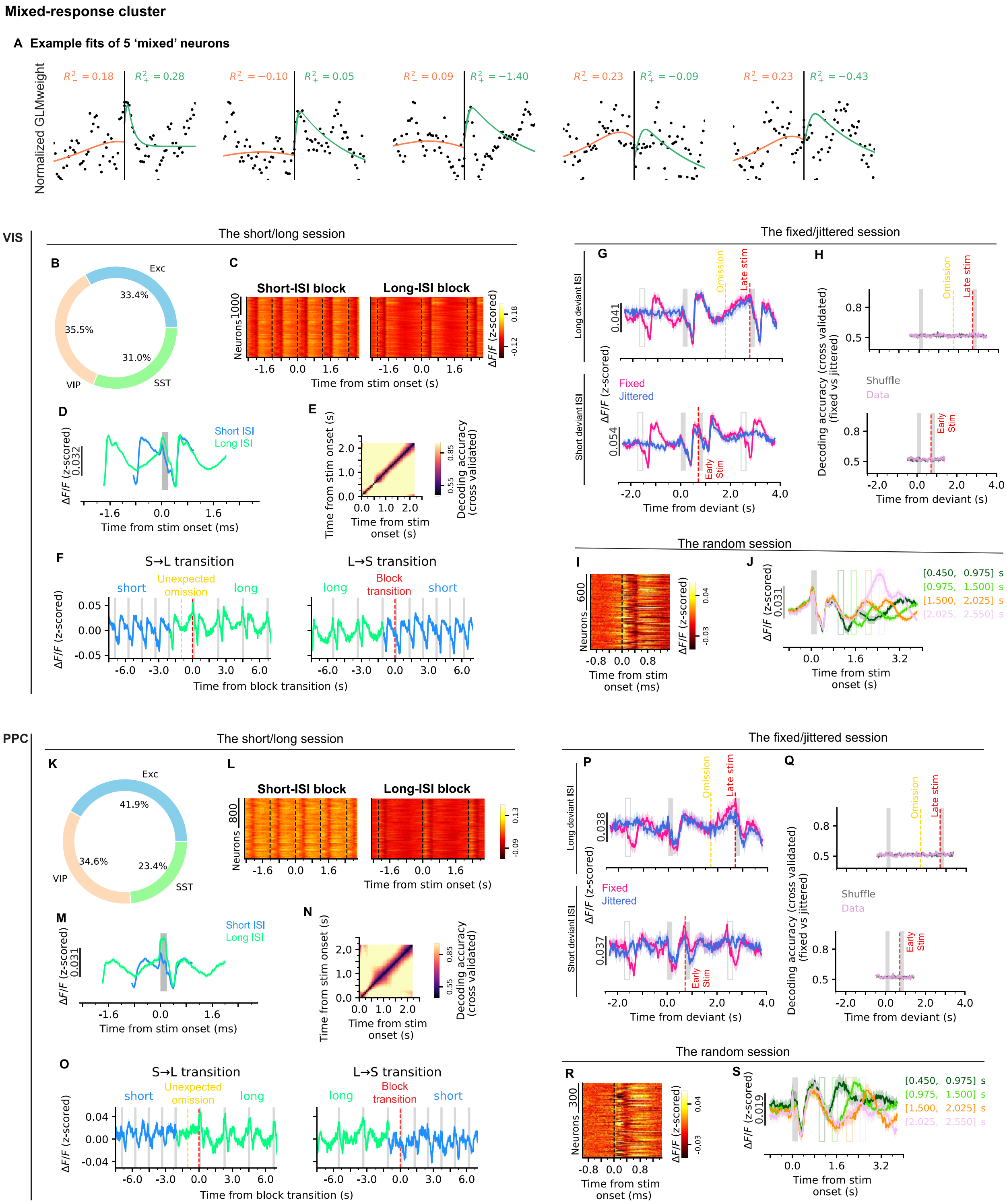

Fig. S3. Neurons in the "mixed" cluster reproduce the main findings across all three paradigms. Activity of neurons lacking exclusively ramp-up or ramp-down dynamics, and therefore excluded from the main figures, is shown across all three paradigms. **VIS:** top panels; **PPC:** bottom panels.

(A) Five examples of GLM kernel components fit by the ramp-up (green) and ramp-down (orange) models. Goodness-of-fit values for both models is indicated. These neurons are grouped in the ‘mixed’ cluster because their goodness-of-fit for neither the ramp-up or down models exceeded our threshold (0.4) determined empirically. Dots: normalized GLM kernel weights centered at time 0 (black vertical line).

(B-F) Data from short/long-interval sessions, demonstrating rapid response changes immediately following block switches.
(B) Fraction of cell types within the “mixed” cluster.
(C) Heatmaps of trial-averaged, single-neuron activity aligned on stimuli (vertical dashed lines) in the short (Left) and long (Right) ISI blocks. Similar to **Fig. 3A**.
(D) Neuron- and trial-averaged activity for short (blue) and long (green) blocks, mean ± 95% confidence interval, aligned on the stimulus (gray box). Similar to **Fig. 3B**.
(E) Pairwise classification accuracy matrices (cross-validated; linear SVM). Each cell shows the accuracy for distinguishing a given pair of time bins. Similar to **Fig. 7B**.
(F) Trial-by-trial neural activity around block transitions, mean ± 95% confidence interval, aligned on the block transition. Similar to **Fig. 4B**.

(G-H) Data from fixed/jittered-interval sessions, demonstrating similar deviant responses—to omissions and early/late stimuli—across fixed and jittered blocks.
(G) Neural responses (mean ± 95% confidence interval) for each cluster, for fixed (red) and jittered (blue) blocks, aligned on the stimulus before the deviant ISI (Top: long deviant ISI; Bottom: short deviant ISI). Similar to **Fig. 5A,E**.
(H) Decoding accuracy for classifying fixed vs. jittered trials (cross-validated; linear SVM; Purple: real data; Gray: shuffled data; Yellow line: omission; Top: long deviant ISI; Bottom: short deviant ISI). Similar to **Fig. 5B,F**.

(I-J) Data from the random-interval sessions, demonstrating the evolution of responses during the ISI.
(I) Heatmap of trial-averaged, single-neuron activity aligned on stimulus onset (vertical dashed line). Similar to **Fig. 6A**.
(J) Random ISIs divided into four bins (different colors). Neural responses averaged across trials within each ISI bin. Similar to **Fig. 6F**.

(K-S) Same as **(B-J)**, but for PPC.

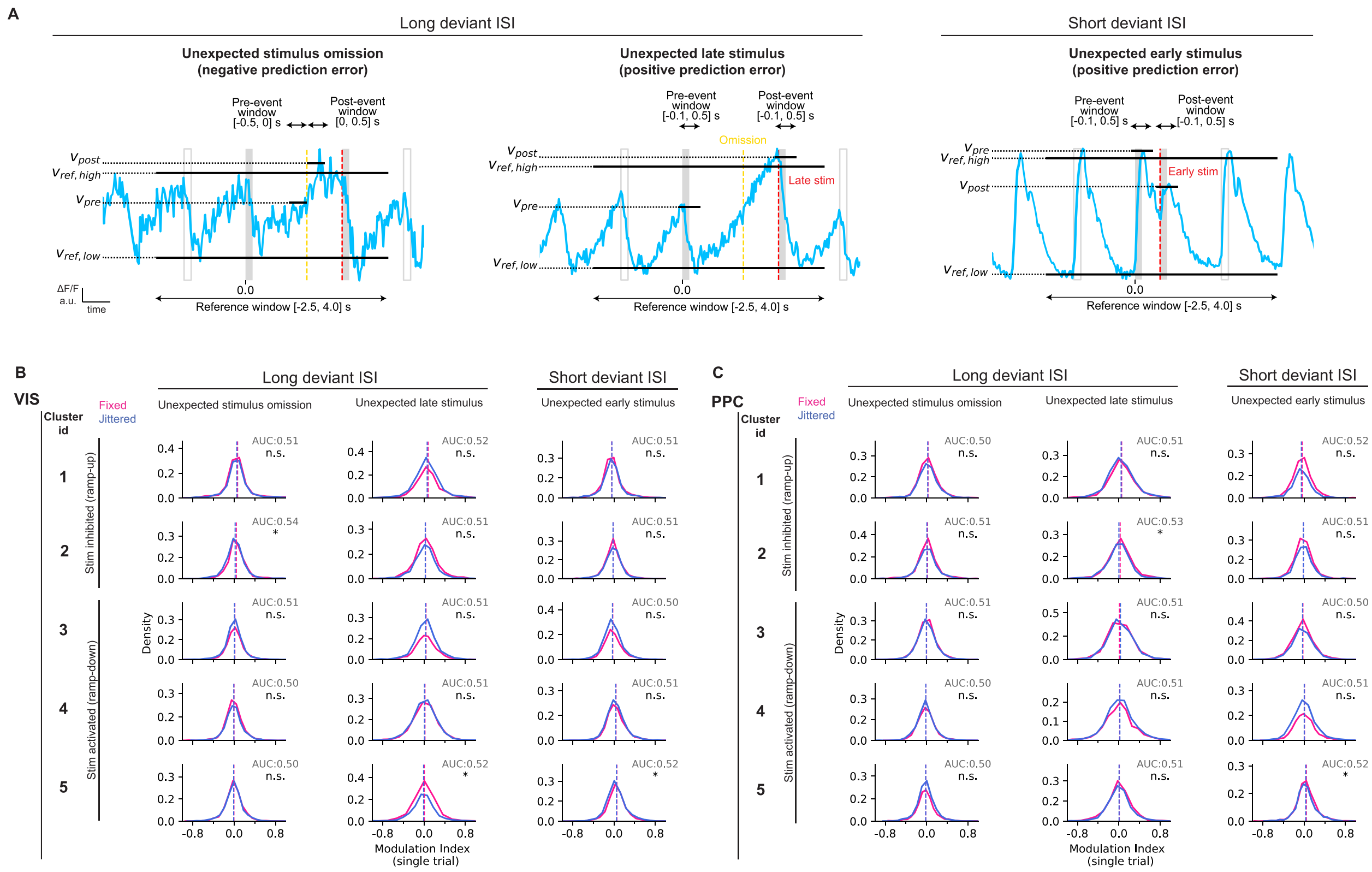

Fig. S4. Responses to temporal deviations are similar in fixed and jittered contexts, arguing against temporal predictive processing as the underlying mechanism of these neuronal dynamics.

(A) Illustration of the modified modulation index with an example. It is computed as $(v_{post} - v_{pre})/(v_{ref,high} - v_{ref,low})$. Solid horizontal lines specify quantification windows. $v_{ref,high}$ and $v_{ref,low}$ are computed from the highest and lowest 10% activity values within the same reference window, respectively. The pre- and post-event windows are distinctly defined for each case (omissions, late stimuli, early stimuli), but the reference windows are the same for all cases.

(B) Modulation indices were computed on single-trial, neuron-averaged responses for each cluster (top to bottom), separately for fixed and jittered trials. Distribution of modulation indices across fixed (pink) and jittered (blue) trials (dashed line: distribution mean). ROC analysis was performed to compare the two distributions. Area under the curve (AUC) values are reported, along with statistical significance (n.s.: non-significant; asterisk: $p < 0.05$). Statistical significance was assessed using a two-sided permutation test (1000 permutations), in which trial labels were randomly shuffled to generate a null distribution of AUC values centered at chance (0.5).

(C) Same as **(B)**, but for PPC.

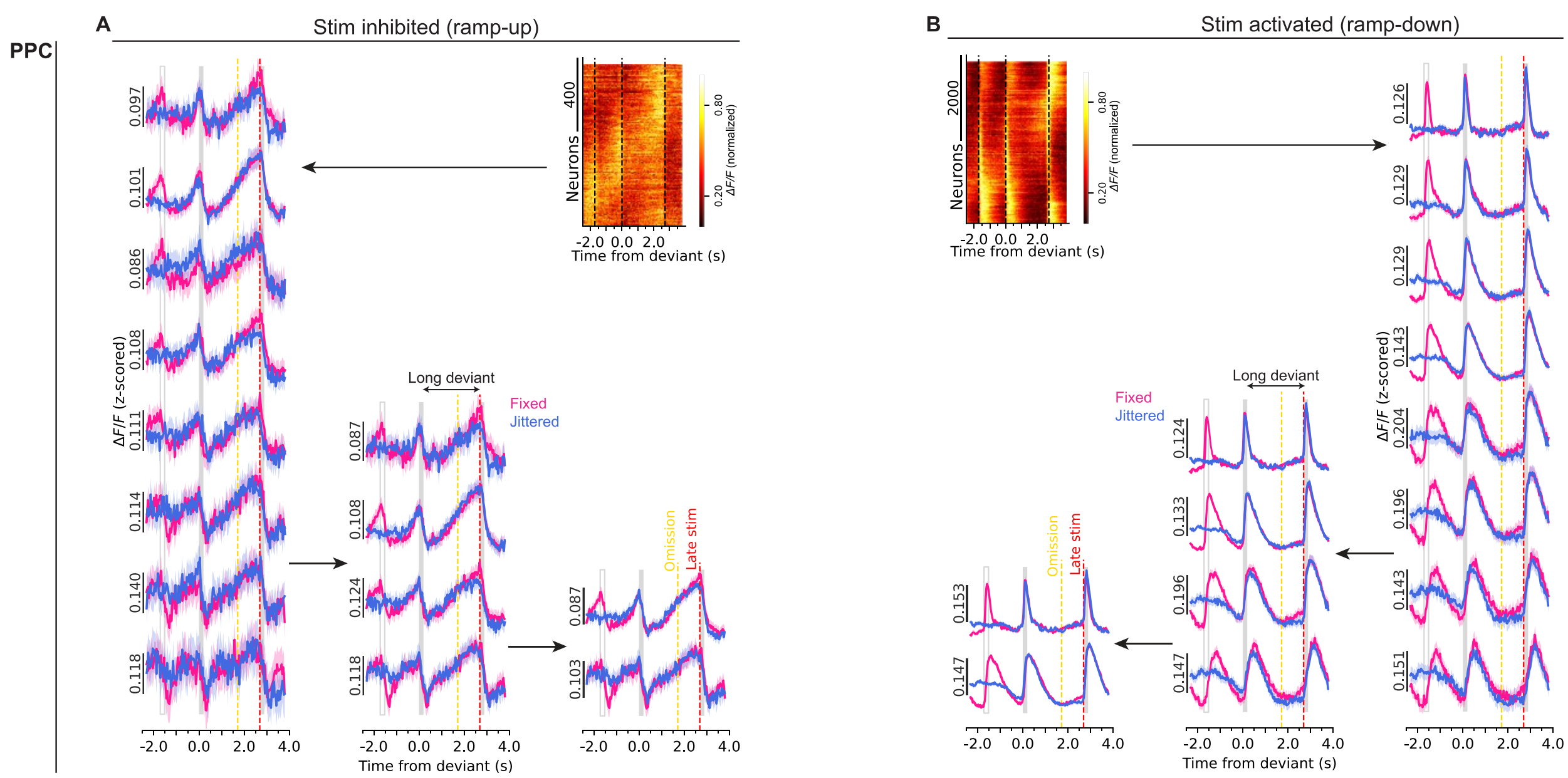

Fig. S5. Intermediate hierarchical subclusters also do not exhibit differential responses between fixed and jittered ISI blocks during long-deviant trials. Data from PPC.

Fixed/jittered-session data during long-deviant ISI trials. Intermediate subclusters are shown throughout the hierarchical merging process for stimulus-inhibited (**A**) and stimulus-activated (**B**) neurons.

Heatmaps show single-neuron trial-averaged activity during fixed long-deviant trials (dashed lines: stimulus onset), sorted using Rastermap (*135*). Arrows indicate the hierarchical merging process from individual neurons to 8, 4, and 2 clusters. Neural responses (mean ± 95% confidence interval) for each cluster are shown for fixed (red) and jittered (blue) blocks, similar to **Fig. 5A,J**.

Average response across the first 10 deviant trials in neurons with high modulation index.

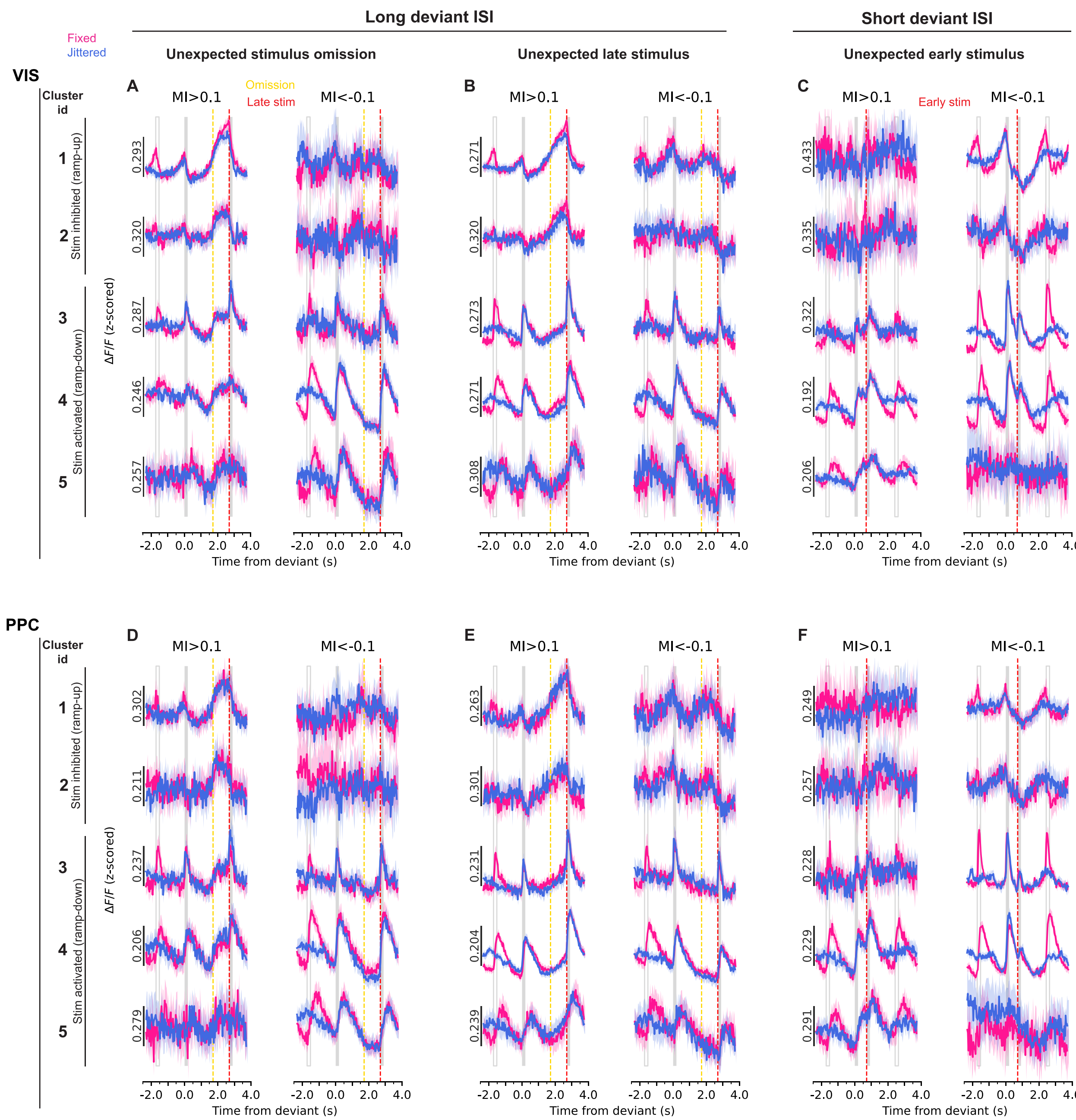

Fig. S6. Selected neurons with high modulation indices still do not exhibit differential responses between fixed and jittered ISI blocks during early deviant trials. VIS: top panels; **PPC:** bottom panels.

Modulation index was computed on trial-averaged activity for individual neurons using quantification windows shown in **Fig. S4A**. Neurons with large modulation indices (above or below 0.1) were selected, and their average activity across the first 10 deviant trials is shown, aligned on omissions during long deviant ISIs **(A)**; late stimuli after long-deviant ISIs **(B)**, and

early stimuli after short-deviant ISIs **(C)**. **Left**: neurons with modulation index < −0.1. **Right**: neurons with modulation index > 0.1.

(D-F) Same as **(A-C)**, but for PPC.

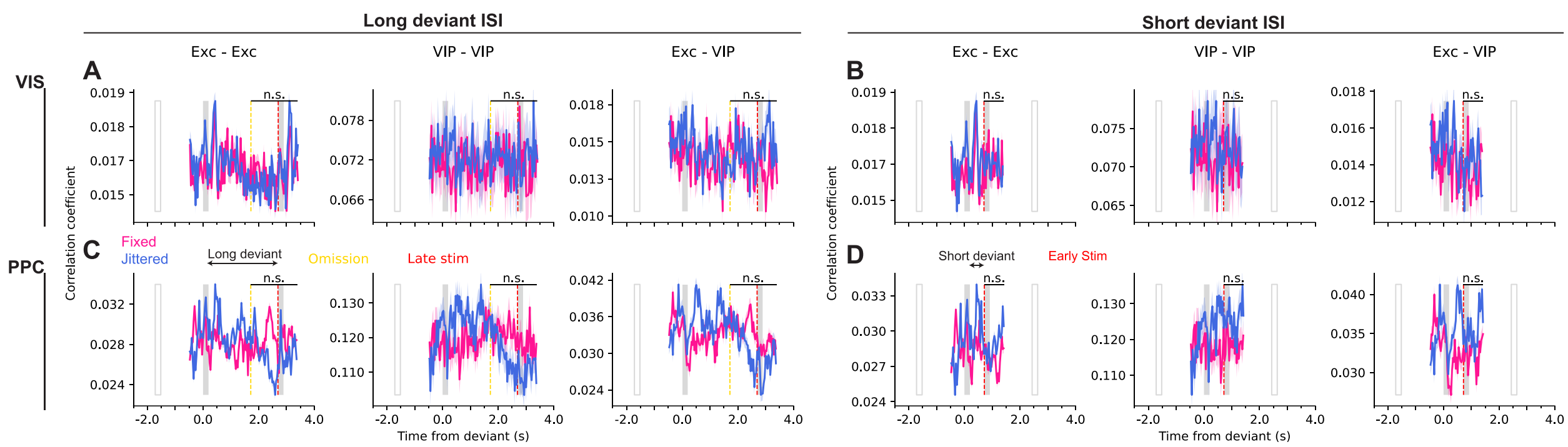

Fig. S7. VIP and excitatory interactions during deviant intervals. VIS: top panels; **PPC:** bottom panels.

Spearman correlation coefficients between simultaneously-imaged excitatory and VIP neurons during long **(A)** and short **(B)** deviant intervals, in fixed/jittered sessions. Traces are aligned on the stimulus before the deviant ISI. For each session, correlation was computed between pairs of neurons across trials, separately for each timepoint within the trial.

Exc-Exc: correlation within excitatory population. **VIP-VIP:** correlation within VIP population. **Exc-VIP:** correlation between excitatory and VIP populations.

Mean ± 95% confidence interval, pooled across all neuron pairs, from all sessions and all mice.

Yellow line: expected but omitted stimulus; Red: unexpected late or early stimulus; Gray boxes: stimuli; Empty boxes: stimuli in fixed and mean stimuli time in jittered). Black lines: statistical test window (n.s.: non-significant; Wilcoxon signed-rank test).

(C-D) Same as **(A-B)**, but for PPC.

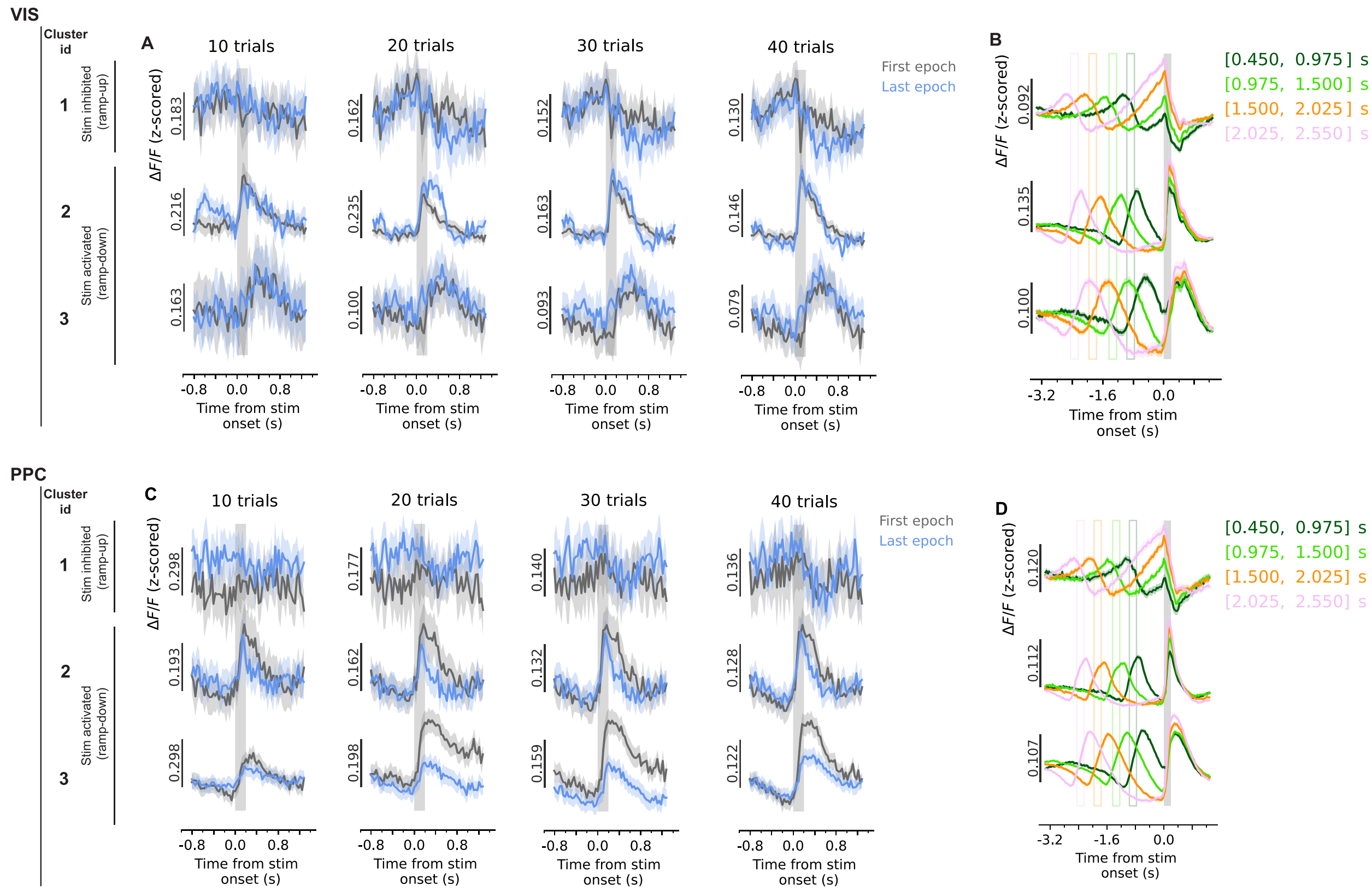

Fig. S8. Responses across the initial trials of the random session confirm that extensive exposure is not required for pre-stimulus ramping activity to emerge. Averaged responses are shown for the first epoch (gray) and last epoch (blue) of the first session. Epochs were defined using different trial numbers to compare response profiles across the beginning and end of the session. Pre-stimulus ramping is evident in VIS in the first 20 trials. Data are noisier in PPC, due to its fewer number of ramp-up neurons (**Fig. 6O**). **VIS:** top panels; **PPC:** bottom panels.

(A) Left to right: averaged responses for the first (gray) and last (blue) 10, 20, 30, 40 trials of the first random session, respectively, similar to **Fig. 6D**.

(B) Random ISIs divided into four bins (different colors). Neural responses averaged across trials within each ISI bin, and aligned to the subsequent stimulus, illustrating larger stimulus-evoked responses following longer preceding ISIs. Gray box: stimulus. Empty boxes: mean ISI of each bin.

(C-D) Same as **(A-B)** but for PPC.

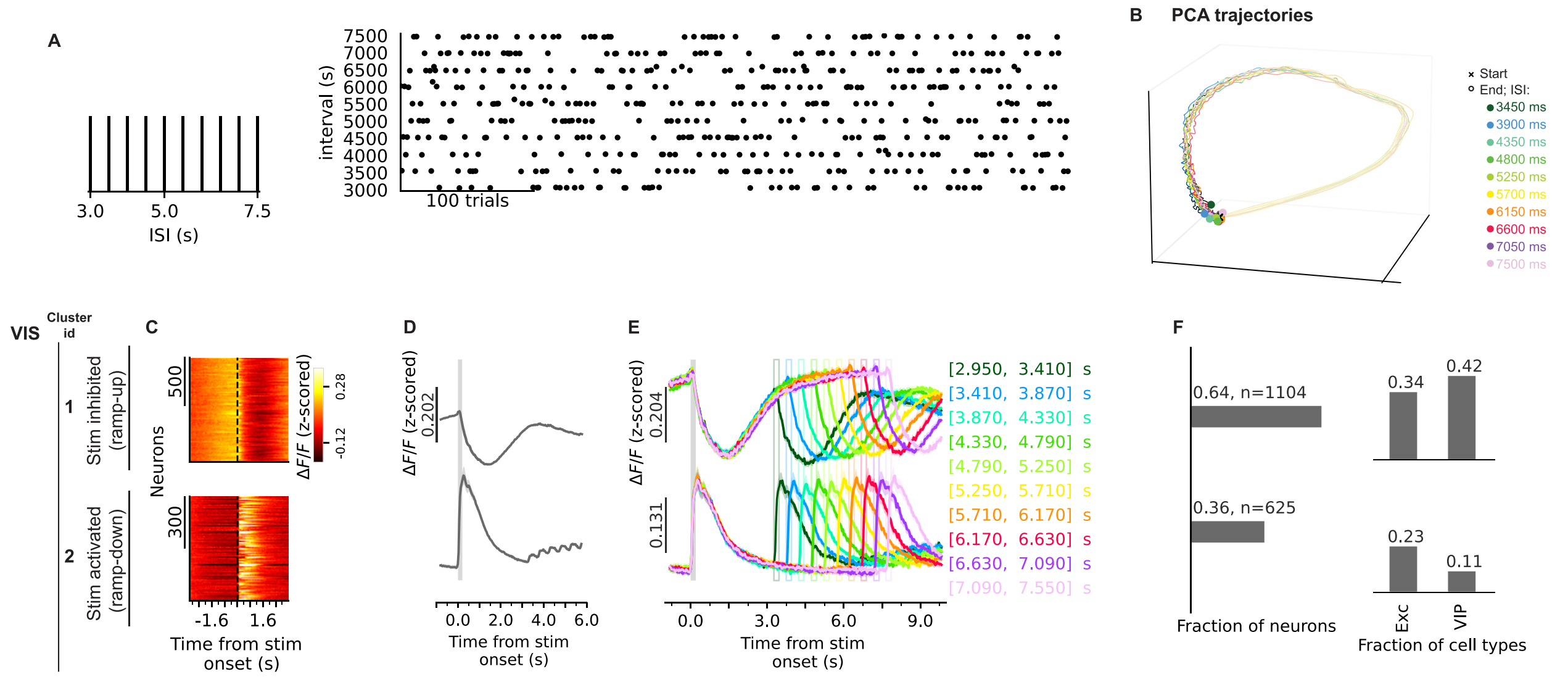

Fig. S9. Ramp-up neural responses plateau about 4s after stimulus onset. Data from extended-ISI random sessions (area VIS; 3 VIP-tdTomato mice; 5 sessions per mouse).

(A) The extended random session is similar to the random session in **Fig. 1H** and **Fig. 6**, but with discrete, uniformly distributed ISIs in the range 3.0–7.5 s. **Left:** distribution of ISIs. **Right:** example sequence of consecutive trial ISIs.

(B) Principal component analysis (top three components) of population activity from stimulus onset through the ISI. Trajectories follow a common manifold across intervals, extending further with longer ISIs.

(C) Heatmaps of trial-averaged, single-neuron activity aligned on stimulus onset (black line), for each cluster (top to bottom).

(D) Neuron- and trial-averaged responses aligned on the stimulus (gray box) for each cluster.

(E) Neural responses averaged across trials within each ISI (different colors). Gray box: stimulus; vertical lines: ISIs.

(F) Left: fraction of all neurons assigned to each cluster. **Right:** fraction of neurons of each cell type assigned to each cluster.

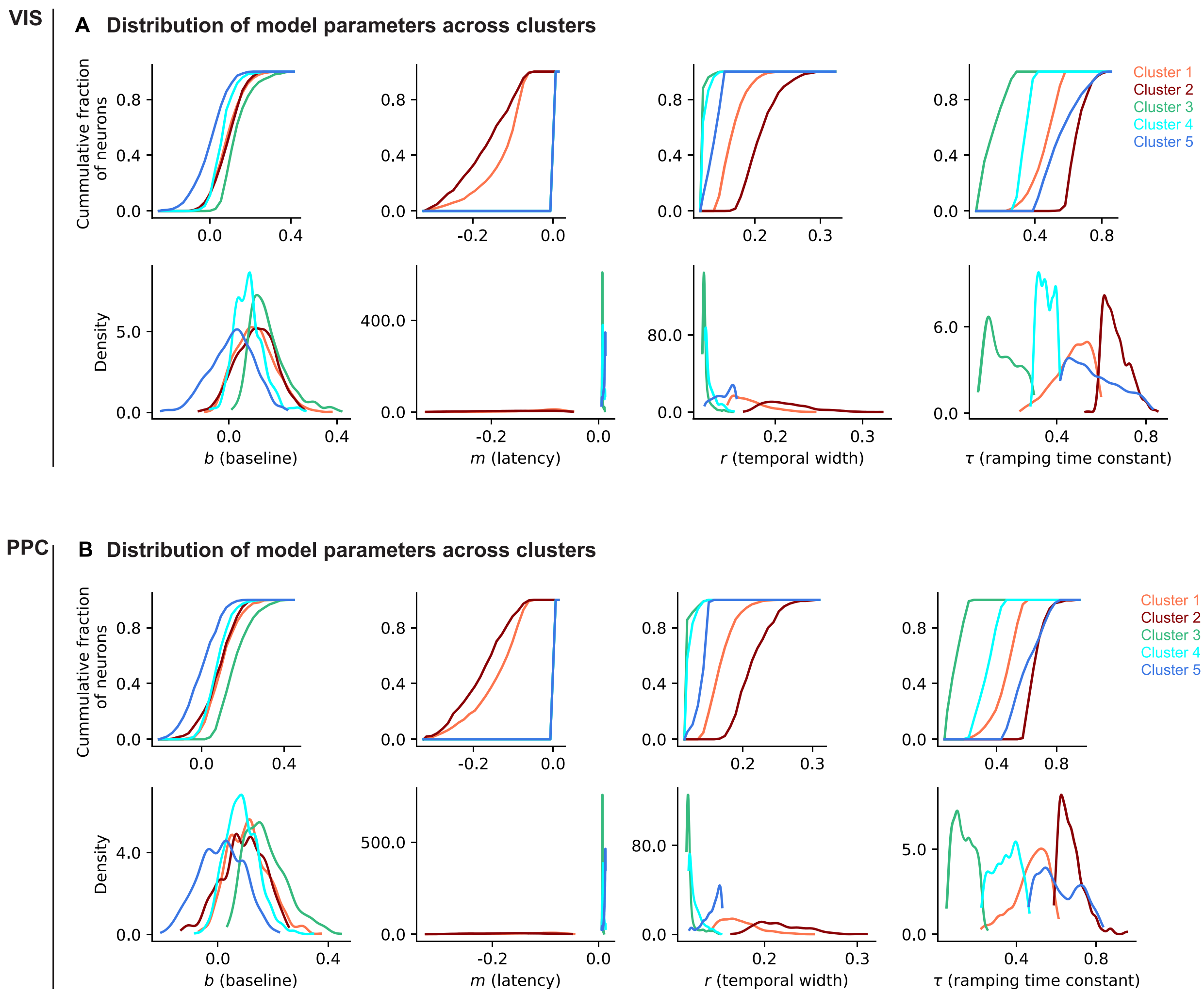

Fig. S10. Distribution of TRF model parameters. Data from the long block of the short/long sessions. Comparison of the 4 parameters of the temporal-receptive-field (TRF) model across all clusters (Orange, red: ramp-up clusters; Green, cyan, blue: ramp-down clusters). **VIS:** top panels; **PPC:** bottom panels.

Top: cumulative histograms. **Bottom:** distribution densities.

'b' reflects the baseline shift.
'm' reflects the time latency of activity peak, relative to stimulus onset.
'τ' is the time constant, reflecting the inverse of ramping speed.
'r' reflects the temporal width of the response.

REFERENCES

1. A. C. Nobre, A. Correa, J. T. Coull, The hazards of time. *Curr. Opin. Neurobiol.* **17**, 465–470 (2007).

2. A. Tsao, S. A. Yousefzadeh, W. H. Meck, M. B. Moser, E. I. Moser, The neural bases for timing of durations. *Nat. Rev. Neurosci.* **23**, 646–665 (2022).

3. J. T. Coull, R.-K. Cheng, W. H. Meck, Neuroanatomical and neurochemical substrates of timing. *Neuropsychopharmacology* **36**, 3–25 (2011).

4. C. J. Cueva, A. Saez, E. Marcos, A. Genovesio, M. Jazayeri, R. Romo, C. D. Salzman, M. N. Shadlen, S. Fusi, Low-dimensional dynamics for working memory and time encoding. *Proc. Natl. Acad. Sci. U.S.A.* **117**, 23021–23032 (2020).

5. N. S. Narayanan, Ramping activity is a cortical mechanism of temporal control of action. *Curr. Opin. Behav. Sci.* **8**, 226–230 (2016).

6. B. J. De Corte, B. Akdoğan, P. D. Balsam, Temporal scaling and computing time in neural circuits: Should we stop watching the clock and look for its gears? *Front. Behav. Neurosci.* **16**, 1022713 (2022).

7. S. Zhou, S. C. Masmanidis, D. V. Buonomano, Neural sequences as an optimal dynamical regime for the readout of time. *Neuron* **108**, 651–658.e5 (2020).

8. J. D. Murray, A. Bernacchia, D. J. Freedman, R. Romo, J. D. Wallis, X. Cai, C. Padoa-Schioppa, T. Pasternak, H. Seo, D. Lee, X.-J. Wang, A hierarchy of intrinsic timescales across primate cortex. *Nat. Neurosci.* **17**, 1661–1663 (2014).

9. J. Wang, D. Narain, E. A. Hosseini, M. Jazayeri, Flexible timing by temporal scaling of cortical responses. *Nat. Neurosci.* **21**, 102–110 (2018).

10. J. J. Paton, D. V. Buonomano, The neural basis of timing: Distributed mechanisms for diverse functions. *Neuron* **98**, 687–705 (2018).

11. V. Mante, D. Sussillo, K. V. Shenoy, W. T. Newsome, Context-dependent computation by recurrent dynamics in prefrontal cortex. *Nature* **503**, 78–84 (2013).

12. C. K. Machens, R. Romo, C. D. Brody, Functional, but not anatomical, separation of "what" and "when" in prefrontal cortex. *J. Neurosci.* **30**, 350–360 (2010).

13. R. Laje, D. V. Buonomano, Robust timing and motor patterns by taming chaos in recurrent neural networks. *Nat. Neurosci.* **16**, 925–933 (2013).

14. D. E. Feldman, The spike-timing dependence of plasticity. *Neuron* **75**, 556–571 (2012).

15. D. V. Buonomano, W. Maass, State-dependent computations: Spatiotemporal processing in cortical networks. *Nat. Rev. Neurosci.* **10**, 113–125 (2009).

16. A. Bernacchia, H. Seo, D. Lee, X. J. Wang, A reservoir of time constants for memory traces in cortical neurons. *Nat. Neurosci.* **14**, 366–372 (2011).

17. E. Pastalkova, V. Itskov, A. Amarasingham, G. Buzsáki, Internally generated cell assembly sequences in the rat hippocampus. *Science* **321**, 1322–1327 (2008).

18. J. F. Medina, M. D. Mauk, Computer simulation of cerebellar information processing. *Nat. Neurosci.* **3**, 1205–1211 (2000).

19. M. S. Matell, W. H. Meck, M. A. Nicolelis, Interval timing and the encoding of signal duration by ensembles of cortical and striatal neurons. *Behav. Neurosci.* **117**, 760–773 (2003).

20. G. B. Mello, S. Soares, J. J. Paton, A scalable population code for time in the striatum. *Curr. Biol.* **25**, 1113–1122 (2015).

21. C. J. MacDonald, K. Q. Lepage, U. T. Eden, H. Eichenbaum, Hippocampal "time cells" bridge the gap in memory for discontiguous events. *Neuron* **71**, 737–749 (2011).

22. D. J. Herzfeld, S. G. Lisberger, Neural circuit mechanisms to transform cerebellar population dynamics for motor control in monkeys. bioRxiv 2025.02.21.639459 [Preprint] (2025). https://doi.org/10.1101/2025.02.21.639459.

23. M. G. Garcia-Garcia, A. Kapoor, O. Akinwale, L. Takemaru, T. H. Kim, C. Paton, A. Litwin-Kumar, M. J. Schnitzer, L. Luo, M. J. Wagner, A cerebellar granule cell-climbing fiber computation to learn to track long time intervals. *Neuron* **112**, 2749–2764.e7 (2024).

24. F. P. Chabrol, A. Arenz, M. T. Wiechert, T. W. Margrie, D. A. DiGregorio, Synaptic diversity enables temporal coding of coincident multisensory inputs in single neurons. *Nat. Neurosci.* **18**, 718–727 (2015).

25. M. I. Leon, M. N. Shadlen, Representation of time by neurons in the posterior parietal cortex of the macaque. *Neuron* **38**, 317–327 (2003).

26. P. Janssen, M. N. Shadlen, A representation of the hazard rate of elapsed time in macaque area LIP. *Nat. Neurosci.* **8**, 234–241 (2005).

27. B. A. Schneider, G. M. Ghose, Temporal production signals in parietal cortex. *PLoS Biol.* **10**, e1001413 (2012).

28. M. J. Hayashi, T. Ditye, T. Harada, M. Hashiguchi, N. Sadato, S. Carlson, V. Walsh, R. Kanai, Time adaptation shows duration selectivity in the human parietal cortex. *PLoS Biol.* **13**, e1002262 (2015).

29. D. Bueti, V. Walsh, The parietal cortex and the representation of time, space, number and other magnitudes. *Philos. Trans. R. Soc. Lond. B Biol. Sci.* **364**, 1831–1840 (2009).

30. J. Kim, J.-W. Ghim, J. H. Lee, M. W. Jung, Neural correlates of interval timing in rodent prefrontal cortex. *J. Neurosci.* **33**, 13834–13847 (2013).

31. M. Xu, S. Y. Zhang, Y. Dan, M. M. Poo, Representation of interval timing by temporally scalable firing patterns in rat prefrontal cortex. *Proc. Natl. Acad. Sci. U.S.A.* **111**, 480–485 (2014).

32. J. P. Gavornik, M. F. Bear, Learned spatiotemporal sequence recognition and prediction in primary visual cortex. *Nat. Neurosci.* **17**, 732–737 (2014).

33. S. Jaramillo, A. M. Zador, The auditory cortex mediates the perceptual effects of acoustic temporal expectation. *Nat. Neurosci.* **14**, 246–251 (2011).

34. Q. Yu, Z. Bi, S. Jiang, B. Yan, H. Chen, Y. Wang, Y. Miao, K. Li, Z. Wei, Y. Xie, Visual cortex encodes timing information in humans and mice. *Neuron* **110**, 4194–4211.e10 (2022).

35. G. B. Keller, T. D. Mrsic-Flogel, Predictive processing: A canonical cortical computation. *Neuron* **100**, 424–435 (2018).

36. K. Friston, S. Kiebel, Predictive coding under the free-energy principle. *Philos. Trans. R. Soc. Lond. B Biol. Sci.* **364**, 1211–1221 (2009).

37. R. P. Rao, D. H. Ballard, Predictive coding in the visual cortex: A functional interpretation of some extra-classical receptive-field effects. *Nat. Neurosci.* **2**, 79–87 (1999).

38. I. Aizenbud, N. Audette, R. Auksztulewicz, K. Basiński, A. M. Bastos, M. Berry, A. Canales-Johnson, H. Choi, C. Clopath, U. Cohen, R. Ponte Costa, R. De Filippo, R. Doronin, S. P. Errington, J. P. Gavornik, C. J. Gillon, A. Granier, J. P. Hamm, L. Hertäg, H. Kennedy, S. Kumar, A. Ladd, H. Ladret, J. A. Lecoq, A. Maier, P. McCarthy, J. Mei, J. Mejias, F. Mikulasch, N. Mudrik, F. Najafi, K. Nejad, H. Nejat, K. Oweiss, M. A. Petrovici, V. Priesemann, L. Rudelt, S. Ruediger, S. Russo, A. Salatiello, W. Senn, E. Sennesh, S. Sima, C. Uran, A. Vasilevskaya, J. Vezoli, M. Vinck, J. A. Westerberg, K. Wilmes, Y. Sophy Xiong, Neural mechanisms of predictive processing: A collaborative community experiment through the OpenScope program. arXiv: 2504.09614 [q-bio.NC] (2025).

39. J. P. Hamm, Y. Shymkiv, S. Han, W. Yang, R. Yuste, Cortical ensembles selective for context. *Proc. Natl. Acad. Sci. U.S.A.* **118**, e2026179118 (2021).

40. A. B. Van Derveer, J. M. Ross, J. P. Hamm, Robust multisensory deviance detection in the mouse parietal associative area. *Curr. Biol.* **33**, 3969–3976.e4 (2023).

41. M. Garrett, S. Manavi, K. Roll, D. R. Ollerenshaw, P. A. Groblewski, N. D. Ponvert, J. T. Kiggins, L. Casal, K. Mace, A. Williford, A. Leon, X. Jia, P. Ledochowitsch, M. A. Buice, W.

Wakeman, S. Mihalas, S. R. Olsen, Experience shapes activity dynamics and stimulus coding of VIP inhibitory cells. *eLife* **9**, e50340 (2020).

42. M. Garrett, P. Groblewski, A. Piet, D. Ollerenshaw, F. Najafi, I. Yavorska, A. Amster, C. Bennett, M. Buice, S. Caldejon, L. Casal, F. D'Orazi, S. Daniel, S. E. d. Vries, D. Kapner, J. Kiggins, J. Lecoq, P. Ledochowitsch, S. Manavi, N. Mei, C. B. Morrison, S. Naylor, N. Orlova, J. Perkins, N. Ponvert, C. Roll, S. Seid, D. Williams, A. Williford, R. Ahmed, D. Amine, Y. Billeh, C. Bowman, N. Cain, A. Cho, T. Dawe, M. Departee, M. Desoto, D. Feng, S. Gale, E. Gelfand, N. Gradis, C. Grasso, N. Hancock, B. Hu, R. Hytnen, X. Jia, T. Johnson, I. Kato, S. Kivikas, L. Kuan, Q. L'Heureux, S. Lambert, A. Leon, E. Liang, F. Long, K. Mace, I. M. d. Abril, C. Mochizuki, C. Nayan, K. North, L. Ng, G. K. Ocker, M. Oliver, P. Rhoads, K. Ronellenfitch, K. Schelonka, J. Sevigny, D. Sullivan, B. Sutton, J. Swapp, T. K. Nguyen, X. Waughman, J. Wilkes, M. Wang, C. Farrell, W. Wakeman, H. Zeng, J. Phillips, S. Mihalas, A. Arkhipov, C. Koch, S. R. Olsen, Stimulus novelty uncovers coding diversity in visual cortical circuits. bioRxiv 2023.02.14.528085 [Preprint] (2023). https://doi.org/10.1101/2023.02.14.528085.

43. F. Najafi, S. Russo, J. Lecoq, Unexpected events trigger task-independent signaling in VIP and excitatory neurons of mouse visual cortex. *iScience* **28**, 111728 (2025).

44. S. Furutachi, A. D. Franklin, A. M. Aldea, T. D. Mrsic-Flogel, S. B. Hofer, Cooperative thalamocortical circuit mechanism for sensory prediction errors. *Nature* **633**, 398–406 (2024).

45. Z. C. Zhou, W. A. Huang, Y. Yu, E. Negahbani, I. M. Stitt, M. L. Alexander, J. P. Hamm, H. K. Kato, F. Fröhlich, Stimulus-specific regulation of visual oddball differentiation in posterior parietal cortex. *Sci. Rep.* **10**, 13973 (2020).

46. A. Fiser, D. Mahringer, H. K. Oyibo, A. V. Petersen, M. Leinweber, G. B. Keller, Experience-dependent spatial expectations in mouse visual cortex. *Nat. Neurosci.* **19**, 1658–1664 (2016).

47. G. Bastos, J. T. Holmes, J. M. Ross, A. M. Rader, C. G. Gallimore, J. A. Wargo, D. S. Peterka, J. P. Hamm, Top-down input modulates visual context processing through an interneuron-specific circuit. *Cell Rep.* **42**, 113133 (2023).

48. N. Nitzan, G. Buzsáki, Diversity of omission responses to visual images across brain-wide regions. *Sci. Adv.* **11**, eadv5651 (2025).

49. S. G. Knudstrup, C. Martinez, J. P. Gavornik, Learned response dynamics reflect stimulus timing and encode temporal expectation violations in superficial layers of mouse V1. eLife13:RP94727 (2025).

50. J. Green, C. A. Bruno, L. Traunmüller, J. Ding, S. Hrvatin, D. E. Wilson, T. Khodadad, J. Samuels, M. E. Greenberg, C. D. Harvey, A cell-type-specific error-correction signal in the posterior parietal cortex. *Nature* **620**, 366–373 (2023).

51. L. Hertäg, H. Sprekeler, Learning prediction error neurons in a canonical interneuron circuit. *eLife* **9**, e57541 (2020).

52. A. J. Keller, M. M. Roth, M. Scanziani, Feedback generates a second receptive field in neurons of the visual cortex. *Nature* **582**, 545–549 (2020).

53. A. Adler, R. Zhao, M. E. Shin, R. Yasuda, W. B. Gan, Somatostatin-expressing interneurons enable and maintain learning-dependent sequential activation of pyramidal neurons. *Neuron* **102**, 202–216.e7 (2019).

54. A. I. Abbas, M. J. M. Sundiang, B. Henoch, M. P. Morton, S. S. Bolkan, A. J. Park, A. Z. Harris, C. Kellendonk, J. A. Gordon, Somatostatin interneurons facilitate hippocampal-prefrontal synchrony and prefrontal spatial encoding. *Neuron* **100**, 926–939.e3 (2018).

55. D. Bueti, The sensory representation of time. *Front. Integr. Neurosci.* **5**, 34 (2011).

56. Y. Zhang, M. Rózsa, Y. Liang, D. Bushey, Z. Wei, J. Zheng, D. Reep, G. J. Broussard, A. Tsang, G. Tsegaye, S. Narayan, C. J. Obara, J.-X. Lim, R. Patel, R. Zhang, M. B. Ahrens, G. C. Turner, S. S. H. Wang, W. L. Korff, E. R. Schreiter, K. Svoboda, J. P. Hasseman, I. Kolb, L. L. Looger, Fast and sensitive GCaMP calcium indicators for imaging neural populations. *Nature* **615**, 884–891 (2023).

57. R. O. Affan, I. M. Bright, L. N. Pemberton, N. A. Cruzado, B. B. Scott, M. W. Howard, Ramping dynamics in the frontal cortex unfold over multiple timescales during motor planning. *J. Neurophysiol.* **133**, 625–637 (2025).

58. R. Cao, I. M. Bright, M. W. Howard, Ramping cells in the rodent medial prefrontal cortex encode time to past and future events via real Laplace transform. *Proc. Natl. Acad. Sci. U.S.A.* **121**, e2404169121 (2024).

59. S. Arroyo, C. Bennett, S. Hestrin, Correlation of synaptic inputs in the visual cortex of awake, behaving mice. *Neuron* **99**, 1289–1301.e82 (2018).

60. D. B. Nestvogel, D. A. McCormick, Visual thalamocortical mechanisms of waking state-dependent activity and alpha oscillations. *Neuron* **110**, 120–138.e4 (2022).

61. P. Avanzini, R. O. Abdollahi, I. Sartori, F. Caruana, V. Pelliccia, G. Casaceli, R. Mai, G. Lo Russo, G. Rizzolatti, G. A. Orban, Four-dimensional maps of the human somatosensory system. *Proc. Natl. Acad. Sci. U.S.A.* **113**, E1936–E1943 (2016).

62. C. Stringer, M. Pachitariu, N. Steinmetz, M. Carandini, K. D. Harris, High-dimensional geometry of population responses in visual cortex. *Nature* **571**, 361–365 (2019).

63. C. Stringer, M. Pachitariu, N. Steinmetz, C. B. Reddy, M. Carandini, K. D. Harris, Spontaneous behaviors drive multidimensional, brainwide activity. *Science* **364**, 255 (2019).

64. M. Jin, L. L. Glickfeld, Magnitude, time course, and specificity of rapid adaptation across mouse visual areas. *J. Neurophysiol.* **124**, 245–258 (2020).

65. A. M. Brands, S. Devore, O. Devinsky, W. Doyle, A. Flinker, D. Friedman, P. Dugan, J. Winawer, I. I. A. Groen, Temporal dynamics of short-term neural adaptation across human visual cortex. *PLoS Comput. Biol.* **20**, e1012161 (2024).

66. B. H. Price, C. M. Jensen, A. A. Khoudary, J. P. Gavornik, Expectation violations produce error signals in mouse V1. *Cereb. Cortex* **33**, 8803–8820 (2023).

67. Q. Zhang, S. Grødem, A. Gracias, K. K. Lensjø, M. Fyhn, C. Stringer, M. Pachitariu, Spatial predictive coding in visual cortical neurons. bioRxiv 2025.09.17.676794 [Preprint] (2025). https://doi.org/10.1101/2025.09.17.676794.

68. S. Ohmae, J. Kunimatsu, M. Tanaka, Cerebellar roles in self-timing for sub- and supra-second intervals. *J. Neurosci.* **37**, 3511–3522 (2017).

69. A. Suvrathan, H. L. Payne, J. L. Raymond, Timing rules for synaptic plasticity matched to behavioral function. *Neuron* **92**, 959–967 (2016).

70. R. Shadmehr, M. A. Smith, J. W. Krakauer, Error correction, sensory prediction, and adaptation in motor control. *Annu. Rev. Neurosci.* **33**, 89–108 (2010).

71. F. Najafi, J. F. Medina, Beyond "all-or-nothing" climbing fibers: Graded representation of teaching signals in Purkinje cells. *Front. Neural Circuits* **7**, 115 (2013).

72. A. Giovannucci, A. Badura, B. Deverett, F. Najafi, T. D. Pereira, Z. Gao, I. Ozden, A. D. Kloth, E. Pnevmatikakis, L. Paninski, C. I. De Zeeuw, J. F. Medina, S. S. Wang, Cerebellar granule cells acquire a widespread predictive feedback signal during motor learning. *Nat. Neurosci.* **20**, 727–734 (2017).

73. J. X. O'Reilly, M. M. Mesulam, A. C. Nobre, The cerebellum predicts the timing of perceptual events. *J. Neurosci.* **28**, 2252–2260 (2008).

74. E. Boven, J. Pickford, R. Apps, N. L. Cerminara, Cerebellar involvement in self-timing. bioRxiv 2025.03.05.641568 [Preprint] (2025). https://doi.org/10.1101/2025.03.05.641568.

75. Z. Gao, C. Davis, A. M. Thomas, M. N. Economo, A. M. Abrego, K. Svoboda, C. I. De Zeeuw, N. Li, A cortico-cerebellar loop for motor planning. *Nature* **563**, 113–116 (2018).

76. F. Najafi, A. Giovannucci, S. S. Wang, J. F. Medina, Sensory-driven enhancement of calcium signals in individual Purkinje cell dendrites of awake mice. *Cell Rep.* **6**, 792–798 (2014).

77. C. D. Tesche, J. J. Karhu, Anticipatory cerebellar responses during somatosensory omission in man. *Hum. Brain Mapp.* **9**, 119–142 (2000).

78. T. Moberget, C. M. Karns, L. Y. Deouell, M. Lindgren, R. T. Knight, R. B. Ivry, Detecting violations of sensory expectancies following cerebellar degeneration: A mismatch negativity study. *Neuropsychologia* **46**, 2569–2579 (2008).

79. M. Kameda, S. Ohmae, M. Tanaka, Entrained neuronal activity to periodic visual stimuli in the primate striatum compared with the cerebellum. *eLife* **8**, e48702 (2019).

80. A. Yaron, T. Shiramatsu-Isoguchi, F. B. Kern, K. Ohki, H. Takahashi, Z. C. Chao, Omission-responsive neurons encode negative prediction error and probability in the auditory cortex. bioRxiv 2024.09.09.612166 [Preprint] (2024). https://doi.org/10.1101/2024.09.09.612166.

81. D. M. Schneider, J. Sundararajan, R. Mooney, A cortical filter that learns to suppress the acoustic consequences of movement. *Nature* **561**, 391–395 (2018).

82. A. Breska, L. Y. Deouell, Neural mechanisms of rhythm-based temporal prediction: Delta phase-locking reflects temporal predictability but not rhythmic entrainment. *PLOS Biol.* **15**, e2001665 (2017).

83. N. Cole, M. Harvey, D. Myers-Joseph, A. Gilra, A. G. Khan, Prediction-error signals in anterior cingulate cortex drive task-switching. *Nat. Commun.* **15**, 7088 (2024).

84. R. Jordan, G. B. Keller, Opposing influence of top-down and bottom-up input on different types of excitatory layer 2/3 neurons in mouse visual cortex. bioRxiv 2020.03.25.008607 [Preprint] (2020). https://doi.org/10.1101/2020.03.25.008607.

85. A. Hockley, L. H. Bohórquez, M. S. Malmierca, Top-down prediction signals from the medial prefrontal cortex govern auditory cortex prediction errors. *Cell Rep.* **44**, 115538 (2025).

86. M. G. Shuler, M. F. Bear, Reward timing in the primary visual cortex. *Science* **311**, 1606–1609 (2006).

87. A. A. Chubykin, E. B. Roach, M. F. Bear, M. G. Shuler, A cholinergic mechanism for reward timing within primary visual cortex. *Neuron* **77**, 723–735 (2013).

88. F. Najafi, G. F. Elsayed, R. Cao, E. Pnevmatikakis, P. E. Latham, J. P. Cunningham, A. K. Churchland, Excitatory and inhibitory subnetworks are equally selective during decision-making and emerge simultaneously during learning. *Neuron* **105**, 165–179.e8 (2020).

89. M. D. Mauk, D. V. Buonomano, The neural basis of temporal processing. *Annu. Rev. Neurosci.* **27**, 307–340 (2004).

90. A. Piet, N. Ponvert, D. Ollerenshaw, M. Garrett, P. A. Groblewski, S. Olsen, C. Koch, A. Arkhipov, Behavioral strategy shapes activation of the Vip-Sst disinhibitory circuit in visual cortex. bioRxiv 2023.04.28.538575 [Preprint] (2023). https://doi.org/10.1101/2023.04.28.538575.

91. K. Rajan, L. F. Abbott, H. Sompolinsky, Stimulus-dependent suppression of chaos in recurrent neural networks. *Phys. Rev. E* **82**, 011903 (2010).

92. G. Hennequin, Y. Ahmadian, D. B. Rubin, M. Lengyel, K. D. Miller, The dynamical regime of sensory cortex: Stable dynamics around a single stimulus-tuned attractor account for patterns of noise variability. *Neuron* **98**, 846–860.e5 (2018).

93. X.-J. Wang, Decision making in recurrent neuronal circuits. *Neuron* **60**, 215–234 (2008).

94. M. M. Churchland, B. M. Yu, J. P. Cunningham, L. P. Sugrue, M. R. Cohen, G. S. Corrado, W. T. Newsome, A. M. Clark, P. Hosseini, B. B. Scott, D. C. Bradley, M. A. Smith, A. Kohn, J. A. Movshon, K. M. Armstrong, T. Moore, S. W. Chang, L. H. Snyder, S. G. Lisberger, N. J. Priebe, I. M. Finn, D. Ferster, S. I. Ryu, G. Santhanam, M. Sahani, K. V. Shenoy, Stimulus onset quenches neural variability: A widespread cortical phenomenon. *Nat. Neurosci.* **13**, 369–378 (2010).

95. H. K. Inagaki, L. Fontolan, S. Romani, K. Svoboda, Discrete attractor dynamics underlies persistent activity in the frontal cortex. *Nature* **566**, 212–217 (2019).

96. S. Wang, R. Falcone, B. Richmond, B. B. Averbeck, Attractor dynamics reflect decision confidence in macaque prefrontal cortex. *Nat. Neurosci.* **26**, 1970–1980 (2023).

97. A. Barri, M. T. Wiechert, M. Jazayeri, D. A. DiGregorio, Synaptic basis of a sub-second representation of time in a neural circuit model. *Nat. Commun.* **13**, 7902 (2022).

98. D. J. Millman, G. K. Ocker, S. Caldejon, I. Kato, J. D. Larkin, E. K. Lee, J. Luviano, C. Nayan, T. V. Nguyen, K. North, S. Seid, C. White, J. Lecoq, C. Reid, M. A. Buice, S. E. J. de Vries, VIP interneurons in mouse primary visual cortex selectively enhance responses to weak but specific stimuli. *eLife* **9**, e55130 (2020).

99. R. H. Masland, P. R. Martin, The unsolved mystery of vision. *Curr. Biol.* **17**, R577–R582 (2007).

100. R. W. Rodieck, Maintained activity of cat retinal ganglion cells. *J. Neurophysiol.* **30**, 1043–1071 (1967).

101. D. M. Piscopo, R. N. El-Danaf, A. D. Huberman, C. M. Niell, Diverse visual features encoded in mouse lateral geniculate nucleus. *J. Neurosci.* **33**, 4642–4656 (2013).

102. H. Shin, H. Adesnik, NDNF interneurons, Spartans of the cortical column: Small in number, strong in impact. *Neuron* **109**, 2041–2042 (2021).

103. R. Tremblay, S. Lee, B. Rudy, GABAergic interneurons in the neocortex: From cellular properties to circuits. *Neuron* **91**, 260–292 (2016).

104. H.-J. Pi, B. Hangya, D. Kvitsiani, J. I. Sanders, Z. J. Huang, A. Kepecs, Cortical interneurons that specialize in disinhibitory control. *Nature* **503**, 521–524 (2013).

105. Y. Fu, M. Kaneko, Y. Tang, A. Alvarez-Buylla, M. P. Stryker, A cortical disinhibitory circuit for enhancing adult plasticity. *eLife* **4**, e05558 (2015).

106. Z. Szadai, H. J. Pi, Q. Chevy, K. Ócsai, D. F. Albeanu, B. Chiovini, G. Szalay, G. Katona, A. Kepecs, B. Rózsa, Cortex-wide response mode of VIP-expressing inhibitory neurons by reward and punishment. *eLife* **11**, e78815 (2022).

107. A. L. Hodgkin, A. F. Huxley, A quantitative description of membrane current and its application to conduction and excitation in nerve. *J. Physiol.* **117**, 500–544 (1952).

108. E. Marder, J. M. Goaillard, Variability, compensation and homeostasis in neuron and network function. *Nat. Rev. Neurosci.* **7**, 563–574 (2006).

109. R. S. Zucker, W. G. Regehr, Short-term synaptic plasticity. *Annu. Rev. Physiol.* **64**, 355–405 (2002).

110. L. F. Abbott, W. G. Regehr, Synaptic computation. *Nature* **431**, 796–803 (2004).

111. S. J. Sara, S. Bouret, Orienting and reorienting: The locus coeruleus mediates cognition through arousal. *Neuron* **76**, 130–141 (2012).

112. J. K. Seamans, C. R. Yang, The principal features and mechanisms of dopamine modulation in the prefrontal cortex. *Prog. Neurobiol.* **74**, 1–58 (2004).

113. M. V. Tsodyks, H. Markram, The neural code between neocortical pyramidal neurons depends on neurotransmitter release probability. *Proc. Natl. Acad. Sci. U.S.A.* **94**, 719–723 (1997).

114. M. Carandini, D. J. Heeger, Normalization as a canonical neural computation. *Nat. Rev. Neurosci.* **13**, 51–62 (2012).

115. J. Benda, A. V. Herz, A universal model for spike-frequency adaptation. *Neural Comput.* **15**, 2523–2564 (2003).

116. M. Beierlein, J. R. Gibson, B. W. Connors, Two dynamically distinct inhibitory networks in layer 4 of the neocortex. *J. Neurophysiol.* **90**, 2987–3000 (2003).

117. Z. Wei, B.-J. Lin, T.-W. Chen, K. Daie, K. Svoboda, S. Druckmann, A comparison of neuronal population dynamics measured with calcium imaging and electrophysiology. *PLOS Comput. Biol.* **16**, e1008198 (2020).

118. C. Bennett, S. Gale, G. Heller, T. Ramirez, H. Belski, A. Piet, O. Zobeiri, A. Amster, A. Arkhipov, A. Cahoon, S. Caldejon, M. Carlson, L. Casal, S. Daniel, C. Farrell, M. Garrett, R. Gillis, C. Grasso, B. Hardcastle, R. Hytnen, T. Johnson, P. Ledochowitsch, Q. L'Heureux, D. Mastrovito, E. McBride, S. Mihalas, C. Mochizuki, C. Morrison, C. Nayan,

K. Ngo, K. North, D. Ollerenshaw, B. Ouellette, P. Rhoads, K. Ronellenfitch, M. Schroedter, J. H. Siegle, C. Slaughterbeck, D. Sullivan, J. Swapp, M. Taormina, W. Wakeman, X. Waughman, A. Williford, J. Phillips, P. A. Groblewski, S. Durand, C. Koch, S. R. Olsen, Map of spiking activity underlying change detection in the mouse visual system. bioRxiv 2025.10.17.683190 [Preprint] (2025). https://doi.org/10.1101/2025.10.17.683190.

119. J. H. Marshel, M. E. Garrett, I. Nauhaus, E. M. Callaway, Functional specialization of seven mouse visual cortical areas. *Neuron* **72**, 1040–1054 (2011).

120. J. Zhuang, L. Ng, D. Williams, M. Valley, Y. Li, M. Garrett, J. Waters, An extended retinotopic map of mouse cortex. *eLife* **6**, e18372 (2017).

121. M. Vélez-Fort, C. V. Rousseau, C. J. Niedworok, I. R. Wickersham, E. A. Rancz, A. P. Brown, M. Strom, T. W. Margrie, The stimulus selectivity and connectivity of layer six principal cells reveals cortical microcircuits underlying visual processing. *Neuron* **83**, 1431–1443 (2014).

122. K. D. Harris, G. M. G. Shepherd, The neocortical circuit: Themes and variations. *Nat. Neurosci.* **18**, 170–181 (2015).

123. E. Maristany de las Casas, K. Killmann, M. Drüke, L. Münster, C. Ebner, R. Sachdev, D. Jaeger, M. E. Larkum, Tuft dendrites in frontal motor cortex enable flexible learning. bioRxiv 2025.03.13.642781 [Preprint] (2025). https://doi.org/10.1101/2025.03.13.642781.

124. E. Abs, R. B. Poorthuis, D. Apelblat, K. Muhammad, M. B. Pardi, L. Enke, D. Kushinsky, D.-L. Pu, M. F. Eizinger, K.-K. Conzelmann, I. Spiegel, J. J. Letzkus, Learning-related plasticity in dendrite-targeting layer 1 interneurons. *Neuron* **100**, 684–699.e6 (2018).

125. L. A. Ibrahim, S. Huang, M. Fernandez-Otero, M. Sherer, Y. Qiu, S. Vemuri, Q. Xu, R. Machold, G. Pouchelon, B. Rudy, G. Fishell, Bottom-up inputs are required for establishment of top-down connectivity onto cortical layer 1 neurogliaform cells. *Neuron* **109**, 3473–3485.e5 (2021).

126. The International Brain Laboratory, V. Aguillon-Rodriguez, D. Angelaki, H. Bayer, N. Bonacchi, M. Carandini, F. Cazettes, G. Chapuis, A. K. Churchland, Y. Dan, E. Dewitt, M. Faulkner, H. Forrest, L. Haetzel, M. Häusser, S. B. Hofer, F. Hu, A. Khanal, C. Krasniak, I. Laranjeira, Z. F. Mainen, G. Meijer, N. J. Miska, T. D. Mrsic-Flogel, M. Murakami, J.-P. Noel, A. Pan-Vazquez, C. Rossant, J. Sanders, K. Socha, R. Terry, A. E. Urai, H. Vergara, M. Wells, C. J. Wilson, I. B. Witten, L. E. Wool, A. M. Zador, Standardized and reproducible measurement of decision-making in mice. *eLife* **10**, e63711 (2021).

127. A. L. Juavinett, I. Nauhaus, M. E. Garrett, J. Zhuang, E. M. Callaway, Automated identification of mouse visual areas with intrinsic signal imaging. *Nat. Protoc.* **12**, 32–43 (2017).

128. S. E. J. de Vries, J. A. Lecoq, M. A. Buice, P. A. Groblewski, G. K. Ocker, M. Oliver, D. Feng, N. Cain, P. Ledochowitsch, D. Millman, K. Roll, M. Garrett, T. Keenan, L. Kuan, S. Mihalas, S. Olsen, C. Thompson, W. Wakeman, J. Waters, D. Williams, C. Barber, N. Berbesque, B. Blanchard, N. Bowles, S. D. Caldejon, L. Casal, A. Cho, S. Cross, C. Dang, T. Dolbeare, M. Edwards, J. Galbraith, N. Gaudreault, T. L. Gilbert, F. Griffin, P. Hargrave, R. Howard, L. Huang, S. Jewell, N. Keller, U. Knoblich, J. D. Larkin, R. Larsen, C. Lau, E. Lee, F. Lee, A. Leon, L. Li, F. Long, J. Luviano, K. Mace, T. Nguyen, J. Perkins, M. Robertson, S. Seid, E. Shea-Brown, J. Shi, N. Sjoquist, C. Slaughterbeck, D. Sullivan, R. Valenza, C. White, A. Williford, D. M. Witten, J. Zhuang, H. Zeng, C. Farrell, L. Ng, A. Bernard, J. W. Phillips, R. C. Reid, C. Koch, A large-scale standardized physiological survey reveals functional organization of the mouse visual cortex. *Nat. Neurosci.* **23**, 138–151 (2020).

129. G. Bastos, J. T. Holmes, J. M. Ross, A. M. Rader, C. G. Gallimore, D. S. Peterka, J. P. Hamm, A frontosensory circuit for visual context processing is synchronous in the theta/alpha band. bioRxiv 2023.02.25.530044 [Preprint] (2023). https://doi.org/10.1101/2023.02.25.530044.

130. M. Pachitariu, C. Stringer, M. Dipoppa, S. Schröder, L. F. Rossi, H. Dalgleish, M. Carandini, K. D. Harris, Suite2p: Beyond 10,000 neurons with standard two-photon microscopy. bioRxiv 061507 [Preprint] (2017). https://doi.org/10.1101/061507.

131. C. Stringer, M. Pachitariu, Cellpose3: One-click image restoration for improved cellular segmentation. *Nat. Methods* **22**, 592–599 (2025).

132. I. M. Bright, M. L. Meister, N. A. Cruzado, Z. Tiganj, E. A. Buffalo, M. W. Howard, A temporal record of the past with a spectrum of time constants in the monkey entorhinal cortex. *Proc. Natl. Acad. Sci. U.S.A.* **117**, 20274–20283 (2020).

133. N. A. Cruzado, Z. Tiganj, S. L. Brincat, E. K. Miller, M. W. Howard, Conjunctive representation of what and when in monkey hippocampus and lateral prefrontal cortex during an associative memory task. *Hippocampus* **30**, 1332–1346 (2020).

134. N. A. Steinmetz, P. Zatka-Haas, M. Carandini, K. D. Harris, Distributed coding of choice, action and engagement across the mouse brain. *Nature* **576**, 266–273 (2019).

135. C. Stringer, L. Zhong, A. Syeda, F. Du, M. Kesa, M. Pachitariu, Rastermap: A discovery method for neural population recordings. *Nat. Neurosci.* **28**, 201–212 (2025).
